# Context-Aware Trust and Reputation Routing Protocol for Opportunistic IoT Networks

**DOI:** 10.3390/s24237650

**Published:** 2024-11-29

**Authors:** Jagdeep Singh, Sanjay Kumar Dhurandher, Isaac Woungang, Han-Chieh Chao

**Affiliations:** 1 Sant Longowal Institute of Engineering and Technology, Longowal 148106, India; jagdeep@sliet.ac.in; 2 Department of Information Technology, Netaji Subhas University of Technology, New Delhi 110078, India; dhurandher@gmail.com; 3 National Institute of Electronics and Information Technology, New Delhi 110078, India; 4 Department of Computer Science, Toronto Metropolitan University, Toronto, ON M5B 2K3, Canada; 5 Department of Applied Informatics, Fo Guang University, Yilan 262307, Taiwan; hcchao@gmail.com; 6 Department of Electrical Engineering, National Dong Hwa University, Hualien 974301, Taiwan; 7 Institute of Computer Science and Innovation, UCSI University, Kuala Lumpur 56000, Malaysia; 8 Department of Artificial Intelligence, Tamkang University, New Taipei City 251301, Taiwan

**Keywords:** opportunistic IoT networks, beta distribution, trust, reputation, security, Opportunistic Network Environment (ONE) simulator

## Abstract

In opportunistic IoT (OppIoT) networks, non-cooperative nodes present a significant challenge to the data forwarding process, leading to increased packet loss and communication delays. This paper proposes a novel Context-Aware Trust and Reputation Routing (CATR) protocol for opportunistic IoT networks, which leverages the probability density function of the beta distribution and some contextual factors, to dynamically compute the trust and reputation values of nodes, leading to efficient data dissemination, where malicious nodes are effectively identified and bypassed during that process. Simulation experiments using the ONE simulator show that CATR is superior to the Epidemic protocol, the so-called beta-based trust and reputation evaluation system (denoted BTRES), and the secure and privacy-preserving structure in opportunistic networks (denoted PPHB+), achieving an improvement of 22%, 15%, and 9% in terms of average latency, number of messages dropped, and average hop count, respectively, under varying number of nodes, buffer size, time to live, and message generation interval.

## 1. Introduction

The integration of short-range communication technologies like WiMAX and Bluetooth [1,2] into portable devices has given rise to a complex network referred to as opportunistic networks (OppNets). In such networks, a fixed path from the source to the destination nodes is not guaranteed. Also, nodes can move freely, and connectivity is unpredictable. To deal with these uncertainties, the so-called store-carry-and-forward mechanism [3] is utilized for data packet transfer, meaning that when a node receives a data packet and no direct path to the destination is available, it holds it until another node is encountered that can carry the packet toward the destination node. These intermediate nodes (also called relay nodes) temporarily store and forward the data packets as they move through the network. Thus, in OppNets, packet forwarding is a cooperative process that depends upon the cooperation and reliability of the relay nodes. Furthermore, since mobile nodes are generally carried by humans, OppNets can take advantage of their social relationships and mobility to connect with them. However, it may happen that some of these nodes do not intentionally cooperate in the message-forwarding process or because of reasons, such as limited buffer and limited battery power. Therefore, one of the major challenges for any routing model in OppNet is to determine suitable and reliable nodes for efficient data forwarding.

Additionally, from a human-centric perspective, analyzing the tight-coupled relationship between humans and the opportunistic connection of smart things has enabled the advent of Opportunistic IoT (OppIoT) networks [4]. In an OppIoT network, the mobility of nodes (i.e., devices carried by humans), along with their inbuilt capability of short-range sensing, are exploited for collecting, routing, and sharing data among human communities, yielding a harmonious interaction between the IoT and humans. In such interaction, some nodes may act erratically by disseminating a lot of insignificant messages or by refusing to forward them, ending by degrading the network performance. On the other hand, some nodes may just accept messages and would not forward them. It would be reasonable for these problematic nodes to be identified and denied participation in the data routing process.

The primary assumption in most of the routing protocols for OppIoT networks is that all the nodes cooperatively and securely carry forward the given message and eventually transmit it to the intended destination. However, in a real-world scenario, it has been observed that although there are several nodes that forward the messages to other nodes, there are some that may not forward the given message due to limited buffer and power. Consequently, the major challenges encountered in routing models for OppIoT stem from the fact that (i) these models do not use a conventional network architecture; (ii) intermittent connectivity between the nodes often occurs at irregular times; and (iii) due to limited battery power, nodes may drain out their energy during the data transmission process, which might lead to severe network failure. In addition to these considerations, an effective security model that can be utilized to detect and isolate the potentially threatening nodes and prevent them from interfering with the packet transmission process is necessary.

### Main Contribution

This paper presents a novel routing protocol (referred to as Context-Aware Trust and Reputation Routing (CATR)) protocol for OppIoT networks, which utilizes the popular beta distribution and node’s context information to compute the trust and reputation of a node. This value is then compared with a specific predefined threshold to determine whether that node is a suitable forwarder or not for the message. In addition, an effective security model based on trust rating and reputation is also proposed, which systematically identifies and isolates those nodes with malicious intent, preventing them from participating in the data forwarding process. The main contributions are as follows.

- This article addresses the network routing issues in OppIoT scenarios, proposing a solution to ensure the stability of the system.
- We propose a Context-Aware Trust and Reputation Routing (*CATR*) protocol and verify its performance using the real CRAWDAD dataset Cambridge/haggle (29 May 2009).
- We have evaluated our proposed CATR routing protocol for OppIoT using an Opportunistic Network Environment (ONE) simulator [5] and compared its performance against that of the Epidemic [6], BTRES [7], and PPHB+ [8] protocols.

The rest of this paper is organized as follows. Section 2 discusses some representative routing protocols for OppIoT networks. Section 3 describes our proposed CATR routing model for OppIoT networks. Section 4 describes the simulation experiments conducted to evaluate the proposed CATR protocol and the results obtained. Finally, Section 5 concludes this paper.

## 2. Related Work

In this section, representative routing schemes for OppIoT networks are discussed [7,8,9,10,11,12,13,14,15,16,17,18,19].

In [6], Vahdat and Becker proposed the Epidemic routing protocol for OppNets, where the eventual message delivery from a source node to a destination node is achieved via random pairwise exchanges of messages among the nodes based on a flooding strategy, minimizing the total resources consumed during message delivery. In such an approach, a summary vector is exchanged among the relay nodes to identify the data packets residing at that buffer. Based on this, a mechanism is then invoked, which enables every data packet to eventually reach every node and get sent to the intended destination. However, since there are multiple copies of the same data packet in the network, this raises a resource consumption issue, which might lead to possible network congestion.

In [9], Dhurandher et al. proposed a method for identifying malevolent nodes within the OppNet ecosystem, which depends on the infrastructure nodes and established cryptography mechanism. The proposed secured algorithm makes use of this cryptographic technique in trust-based routing for guaranteeing the participation of more nodes in the routing process, by building confidence in their participation. Simulation experiments are conducted, and confirming the effectiveness of the proposed scheme can enhance the network performance in terms of the considered performance metrics. While the proposed method effectively identifies malevolent nodes and enhances network performance through cryptographic techniques in trust-based routing, it relies heavily on infrastructure nodes and an established cryptographic framework. This dependency could limit its applicability in fully decentralized or resource-constrained OppNet environments where infrastructure support and computational resources for cryptographic operations are not readily available.

In [10], Naveena et al. proposed a trust-based routing scheme for OppNets, which consists of two steps: data retrieval and data transfer. The data retrieval step is meant to identify and then maintain each node’s data transfer, whereas the data transfer step is meant to process the prediction of a safe path to be used for the transmission of the data packets to the destination node. With this strategy, nodes do, however, consume a lot of energy. This strategy involves significant energy consumption by nodes during the data retrieval and transfer steps, which may limit its practicality in energy-constrained environments such as mobile or battery-powered devices.

In [11], Elmahdi et al. proposed a modified multi-path distance vector (MMDV) protocol for OppNets and designed a trustworthy and secure mechanism for transmitting the data amid a blackhole threat. In their approach, the messages are split up into several paths to get to their destinations, and the transmission of the messages is secured using a homomorphic encryption algorithm. Simulation results are provided, validating the effectiveness of the MMDV protocol in terms of packet delivery and network throughput. The splitting of messages into multiple paths increases computational and communication overhead, which may pose challenges in resource-constrained OppNet environments. Additionally, the reliance on homomorphic encryption could introduce delays due to its computational intensity.

In [7], Fang et al. proposed a trust reputation evaluation mechanism for wireless sensor networks (referred to as a beta-based Trust and Reputation Evaluation System (BTRES), which can be applied to OppNets. Such a mechanism relies on the use of the beta distribution to simulate the reputation of a node after monitoring its behavior, and then calculate its trust value and credibility. The trust values of nodes are then utilized to guide the selection of appropriate nodes that participate in the message-routing process. Simulation experiments are conducted, validating the effectiveness of the proposed mechanism in enhancing the information security in the network, and in defending against the internal attacks that originate from those nodes that are compromised. The reliance on beta distribution modeling requires extensive monitoring and trust calculations, which can lead to increased computational and communication overhead. Additionally, the approach may struggle in dynamic or highly mobile OppNet environments where node behaviors change rapidly, potentially affecting the accuracy of trust evaluations.

In [8], Rashidibajgan et al. proposed a routing method for OppNets, which also secures the network against dropping and Sybil attacks. Its design consists of a trust structure for ensuring the confidentiality of messages, a shared public-key approach for data integrity and privacy protection purposes, and a cooperative mechanism to resist selfish nodes. Through experiments, the proposed scheme is shown to protect against selective dropping attacks and Sybil attacks while yielding a better performance in terms of message delivery ratio and average latency compared to that yielded by selected benchmark schemes.  The reliance on a shared public-key approach and cooperative mechanisms may introduce computational overhead and increase energy consumption, making it less suitable for resource-constrained devices. Additionally, the method’s performance in highly dynamic or large-scale OppNet environments may require further evaluation to ensure scalability and adaptability.

In [13], Wang et al. proposed a dynamic trust framework for opportunistic mobile social networks, which enable a given node to determine the trust of another node by relying on the behavior of the latter. Such a trust model is designed using a two-hop feedback method based on three parameters, namely fitness, connectedness, and satisfaction, which help quantify the honesty of a node. Through simulations, the proposed trust architecture is shown to effectively identify abnormal nodes, as well as nodes that have launched conspiracy attacks. The reliance on indirect feedback and predefined parameters may reduce its accuracy in rapidly changing network conditions or environments with sparse connectivity. Additionally, the computational complexity of the two-hop feedback mechanism could be a limitation for nodes with constrained resources.

In [14], Li et al. proposed a trust-based secure routing algorithm for OppNets, in which a trust value for each node is calculated by gathering the message’s delay to the destination node and a packet-forwarding evidence chain. The values are maintained locally by each node in a vector table. This vector table is then broadcasted periodically, along with a signature and timestamp, leading to the construction of a trust routing table that is hosted at each node, which then helps in determining a suitable next-hop relay node to forward the message toward its intended destination node. Experiments are conducted, showing the effectiveness of the proposed algorithm in terms of delivery probability ratio. The periodic broadcasting of vector tables with signatures and timestamps increases communication overhead, potentially leading to higher energy consumption and network congestion in resource-constrained environments. Furthermore, the reliance on locally maintained trust values may limit the algorithm’s adaptability in highly dynamic or large-scale OppNet scenarios.

In [15], Su et al. proposed a trust-based routing scheme for OppNets, where the trust value of a node is calculated by combining Direct and Indirect Trust. Low-trust nodes are then filtered and excluded from participation in the routing process. The considered trust model employs pruning and filtering processes to eliminate harmful proposals. Additionally, by merging the node trust values with the expected transmission count data, an opportunistic routing strategy based on the aforementioned trust model is proposed. Through simulations, the effectiveness of the proposed protocol is demonstrated. The pruning and filtering processes may introduce computational overhead, potentially impacting the protocol’s scalability in large networks. Additionally, the reliance on accurate trust evaluations might make the scheme vulnerable to scenarios with incomplete or inconsistent trust information, limiting its effectiveness in highly dynamic OppNet environments.

In [16], Bangotra et al. proposed a trust-based and secured opportunistic routing scheme for wireless sensor networks, in which the trust value of a node is calculated based on specific parameters, which are the sincerity of the node in forwarding the data packet, sincerity of the node in acknowledging the data packet receipt, and the node’s energy depletion value. Based on this trust value calculation, a relay selection algorithm is invoked to determine the suitability of the considered to participate in the routing process. Through experiments, the performance of the proposed routing protocol is shown to be superior to that of selected trust-based routing schemes in terms of predefined metrics. The protocol’s effectiveness depends on accurate trust evaluations, which could be compromised in the presence of incomplete or misleading data, potentially impacting its robustness in hostile environments.

In [17], Xiao Cai et al. address the communication security challenges in T-S fuzzy network control systems (NCSs) caused by network congestion and denial-of-service (DoS) attacks under a QoS framework. To ensure system stability, the authors propose an intelligent event-triggered controller (IETC) featuring an improved data compression mechanism and a mini-batch gradient descent algorithm to optimize trigger thresholds for bandwidth efficiency. The study also introduces asymmetric Lyapunov–Krasovskii functions (LKFs) to reduce decision variables and enhance control robustness. The effectiveness of the IETC is validated on a CarSim–Simulink platform in an autonomous vehicle system, demonstrating its practical applicability and reliability. The main limitation of this work lies in the reliance on the CarSim–Simulink platform for validation, which may not fully capture the complexities and variations of real-world autonomous vehicle systems or large-scale networks. Additionally, while the IETC approach improves bandwidth efficiency, its performance could degrade in highly dynamic or unpredictable network environments, where the assumptions of stable conditions or accurate model parameters might not hold.

In [18], Sreenivasa et al. proposed a social context-aware routing protocol for OppNets, in which a mapping technique to analyze the recent relationships among two encountered nodes is designed. Based on these, a dynamic intra-cluster mechanism is invoked to reduce the message copies, and an inter-cluster communication method is devised to route the data packets to their intended destinations while eliminating unnecessary message flooding in the network. Simulation experiments have shown the superiority of the proposed scheme against a few benchmark schemes in terms of predefined performance metrics, under varying practical scenarios. The limitation of this work lies in its reliance on the assumption that the social relationships between nodes can be accurately mapped and remain stable over time. In highly dynamic or unpredictable environments, such assumptions may not hold, leading to suboptimal routing decisions. Furthermore, the approach may not be scalable in large networks with a high number of nodes, as managing dynamic intra- and inter-cluster communications could incur significant overhead.

In [19], the author proposed a probabilistic routing model for OppNets, which relies on the nodes’ meeting probabilities, last meeting time, and acknowledgment tables to determine their likelihood to carry the data packets toward their intended destination. In doing so, cross-layer optimization strategies are also designed to reduce the energy consumption of nodes while increasing the message delivery probability. Through experiments, the proposed scheme is shown to be superior to chosen benchmark schemes in terms of packet delivery probability. A limitation of the model is its dependence on accurate estimation of meeting probabilities and last meeting times, which may not always be reliable in highly dynamic and unpredictable environments. Additionally, while the model aims to reduce energy consumption, the computational complexity involved in maintaining acknowledgment tables and performing cross-layer optimization may lead to increased overhead, especially in large-scale networks with numerous nodes. This could negatively impact the overall network performance in terms of scalability and responsiveness.

Unlike the discussed works, this paper proposes a routing model for OppIoT networks, which utilizes the beta distribution and node’s context information to calculate the trust and reputation of every node. The values are then utilized to determine the suitable nodes to carry the data packets from source to destination nodes while isolating those nodes that have malicious intent.

## 3. Proposed CATR Routing Protocol for OppIoT Networks

The design of our CATR protocol relies on the utilization of the beta distribution and the use of the node’s context information to calculate the overall trust value of any node (which involves the calculation of its reputation). These values then serve to determine suitable non-malicious relay nodes that participate in the message routing process from source to destination nodes.

**Figure 1:**
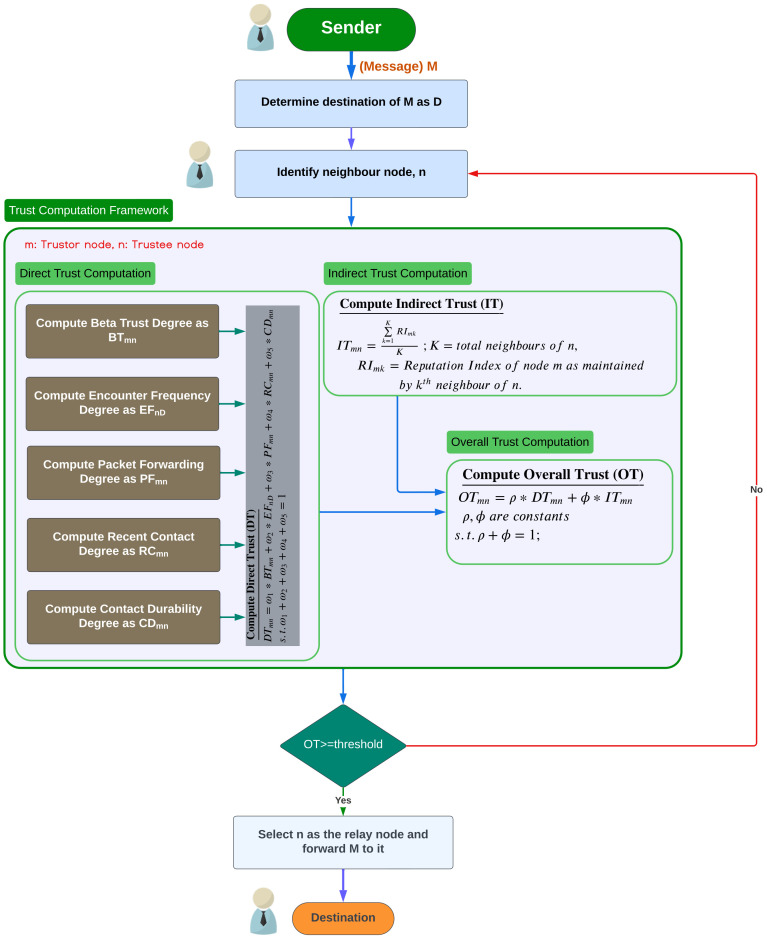
Working flow of our proposed CATR protocol.

The operational flow of our proposed CATR protocol is illustrated in Figure 1, which consists of (a) the Direct Trust (DT) Computation Module—which calculates the DT value of any node, (b) the Indirect Trust (IT) Computation Module—which calculates the Indirect Trust value of any node, and (c) the Overall Trust Computation Module—which aggregates the Direct Trust and Indirect Trust values of any node. We can describe this more precisely as follows:

- The Direct Trust (DT) Computation Module involves five main sub-modules, namely (1) the Computation of Beta Trust Degree sub-module, (2) the Computation of Encounter Frequency Degree sub-module, (3) the Computation of Packet Forwarding Degree sub-module, (4) the Computation of Recent Contact Degree sub-module, and (5) the Computation of Contact Durability Degree sub-module;
- The Indirect Trust (IT) Computation Module calculates the IT value of any node based on the computation of its reputation index (RI), considering the reputations of that node gathered from its neighbors;
- The Overall Trust Computation Module computes the total trust value of any node.

In this work, the calculation of trust value for a given node relies on the beta distribution and the design of a trust mechanism by mimicking the ideas inherited from [12]. The beta distribution is a type of probability distribution for possible values of a probability, which can be involved in the computation of the trust and reputation of a node in a network environment [12]. Through it, one can describe the distribution of the nodes’ credibility by monitoring the nodes’ behavior as completed [7]. This paper makes use of it jointly with a method to calculate the Direct and Indirect Trust of any node in order to determine its trustworthiness. The trust values of nodes are then used in deciding on the selection of relay nodes that are suitable to carry the data packets from their source node to the intended destination node.

### 3.1. Beta Distribution and Trust Modeling

According to the beta distribution [12], the probability density function (PDF) is formulated by using two parameters (α and β) and the gamma function [7], as follows:   

(1) $$P\left(x\right)=f\left(x|\alpha ,\beta \right)=\frac{\Gamma (\alpha +\beta )}{\Gamma \alpha \;\cdot \;\Gamma \beta }x^{\alpha -1}{\left(1-x\right)}^{\beta -1}$$

where 0≤x≤1, α≥0, β≥0, such that if α<1, then x≠0 and if β<1, x≠1. The probability expectation value of this beta distribution is then computed as follows:

(2) $$E\left(x\right)=\frac{\alpha }{\alpha +\beta }$$

In the context of data transmission in OppNet, α and β can represent the successful (resp. unsuccessful) encounters among the nodes. Additionally, let ζ be the number of times that successful encounters have occurred and λ be the number of times that unsuccessful encounters have occurred. Then, the probability density function (PDF) of α, the future can be expressed by setting

(3) α=ζ+1,β=λ+1suchthatζ,ζ≥0

Substituting these values in Equation (1) yields

(4) $$\begin{array}{ccc} P(x) & = & f(x|\alpha ,\beta )\\ & = & f(x|\zeta +1,\lambda +1)\\ & = & \frac{\Gamma (\zeta +1+\zeta +1)}{\Gamma (\zeta +1)\;\cdot \;\Gamma (\lambda +1)}x^{\zeta +1-1}{\left(1-x\right)}^{\lambda +1-1}\\ & = & \frac{\Gamma (\zeta +\lambda +2)}{\Gamma (\zeta +1)\;\cdot \;\Gamma (\lambda +1)}x^{\zeta }{\left(1-x\right)}^{\lambda } \end{array}$$

The corresponding probability expectation in Equation (2) can then be rewritten as

(5) $$\begin{array}{ccc} E(x) & = & \frac{\zeta +1}{\left(\zeta +1)+(\lambda +1\right)}\\ & = & \frac{\zeta +1}{\left(\zeta +\lambda +2\right)} \end{array}$$

This Equation (5) can be used to represent the beta trust of a node *n* as computed by another node *m* (denoted $BT_{mn}$). Additionally, the beta distribution [12] can also be used to determine the reputation index of a node by another node *m* (denoted $RI_{mn}$). Indeed, let *i* be any neighbor of node *n*, $\zeta_{n}^{i}$ be the number of successful encounters between nodes n and *i* as maintained by node *i*, and $\lambda_{n}^{i}$ be the number of unsuccessful encounters between nodes n and *i* as maintained by node *i*. Then, for calculating the reputation of a node, the probability density function (PDF) that can be used (according to Equation (4) is

(6) $$\begin{array}{ccc} P(x) & = & \frac{\Gamma (\zeta_{n}^{i}+\lambda_{n}^{i}+2)}{\Gamma \left(\zeta_{n}^{i}+1\right)\;\cdot \;\Gamma \left(\lambda_{n}^{i}+1\right)}x^{\zeta_{n}^{i}}{\left(1-x\right)}^{\lambda_{n}^{i}} \end{array}$$

such that $\zeta_{n}^{i},\lambda_{n}^{i}\geq 0$. In this case, the probability expectation using Equation (6) is obtained as

(7) $$\begin{array}{ccc} E(x) & = & \frac{\zeta_{n}^{i}+1}{\left(\zeta_{n}^{i}+1\right)+\left(\lambda_{n}^{i}+1\right)}\\ & = & \frac{\zeta_{n}^{i}+1}{\left(\zeta_{n}^{i}+\lambda_{n}^{i}+2\right)} \end{array}$$

This Equation (7) can be utilized to represent the reputation index ($RI_{mn}$) of a node n as computed by node *m*. Based on this, the reputations obtained from those nodes that node n had encountered can be used to calculate its Indirect Trust (*IT*) value of *n*, precisely as the mean value of these reputation values.

For trust modeling purposes, the social status of a node mainly depends upon the trustworthiness and cooperation that it provides to other nodes in the network. This further enhances a belief system among the nodes which can be referred to as trust. Thus, trust can be defined as a measure of belief that one node has in another node; it is influenced by the cooperative behavior among the nodes. For instance, whenever a pair of nodes $N_{1}$ and $N_{2}$ encounter each other, $N_{2}$ can be considered as cooperative and trustworthy for $N_{1}$ if $N_{2}$ can successfully forward a data packet that it received coming from $N_{1}$ without altering its content. However, if node $N_{2}$ fails to forward the received data packet, it is considered as non-cooperative or as having malicious intent. Therefore, node $N_{1}$ must first determine whether $N_{2}$ can be trusted or not. Another possibility is that node $N_{2}$ may present itself as a genuine node; however, upon receiving the data packet, it may drop it. Hence, the considered trust mechanism should not only take into account the behavior of $N_{2}$ to determine whether it is trustworthy or not, but it should also consider the recommendations for node $N_{2}$ that have been received from other nodes. These recommendations are taken into account in the design of our CATR model illustrated in Figure 1.

Using the above-discussed preliminaries, we now focus on the details of the design of the aforementioned modules/sub-modules. The considered notations and hashmap data structure used in this design are given in Table 1 and Table 2, respectively.

### 3.2. Direct Trust Computation

A node’s Direct Trust (DT) is established by its actions within the network. As pointed out earlier in Section 3, its value is obtained as a weighted-average function with five parameters, namely the Beta Trust Degree of a node (BT) obtained from the Computation of Beta Trust Degree sub-module, the Encounter Frequency Degree (EF) obtained from the Computation of Encounter Frequency Degree sub-module, the Packet Forwarding Degree (PF) of a node obtained from the Computation of Packet Forwarding Degree sub-module, the Recent Contact Degree (RD) of a node obtained from the Computation of Recent Contact Degree sub-module, and the Contact Durability Degree (CD) of a node obtained from the Computation of Contact Durability Degree sub-module. Therefore, the DT value $DT_{mn}$ is computed dynamically each time that a node n (referred to as Trustee) has a packet to transmit encounters to another node m (referred to as Trustor) during its movement, and it is obtained as follows:

(8) $$\begin{array}{ccc} DT_{mn} & = & f(BT,EF,PF,RC,CD)\\ & = & \omega_{1}*BT+\omega_{2}*EF+\omega_{3}*PF+\omega_{4}*RC+\omega_{5}*CD \end{array}$$

where

- $\omega_{k};\forall k$ are constants such that $\sum_{k=1}^{5}\omega_{k}=1$.
- BT is computed based on successful and unsuccessful encounters as
  (9) $$BT_{mn}=\frac{se+1}{\left(se+ue+2\right)}$$
  where node *n* is the Trustee and node *m* is the Trustor, se is the number of successful encounters, and ue is the number of unsuccessful encounters. The procedure to calculate $BT_{mn}$ is given in Algorithm 1.
- EF calculation: Whenever any node *m* carrying a data packet encounters a neighbor node *n*, it determines $EF_{nD}$—the encounter frequency degree of *n* with the destination node *D*—which is computed as
  (10) $$\begin{array}{ccc} EF_{nD} & = & \frac{Encounter\;frequency\;of\;n\;with\;D}{Encounter\;frequency\;of\;n\;with\;all\;other\;nodes\;} \end{array}$$
  (11) $$\begin{array}{ccc} EF_{nD} & = & \frac{f_{nD}}{\sum_{k=1}^{K}f_{nk}} \end{array}$$
  where *K* is the total number of nodes encountered by node *n* until that instance of time, $f_{nD}$ is the encounter frequency of *n* with *D*, and $f_{nk}$ is the encounter frequency of *n* with each of the *K* number of nodes. Typically, EF is understood as the ratio of the number of encounters between *n* and *D* to the frequency of contacts that *n* has had with other nodes. Node *n* is comparatively more suited to forward data packets toward node *D*, as indicated by its greater ET value. The procedure to calculate $EF_{nD}$ is given in Algorithm 2.
- PF calculation: PF is computed by a node *m* whenever it encounters any neighbor *n* , as follows:
  (12) $$PF_{mn}=\frac{num_{mn}}{num_{n}}$$
  where $num_{mn}$ is the number of *m* packets forwarded by node *n* and $num_{n}$ is the total number of packets forwarded by node *n* to all other nodes. The procedure to calculate $PF_{mn}$ is given in Algorithm 3.
- RC calculation: RC refers to the latest contact time between two nodes. It is computed based on the time elapsed since the last contact between a node *m* with its neighbor *n* and that between *n* with the destination *D*. Its lower value indicates that these nodes have been contacted more recently. It is represented by $RC_{mn}$, computed as
  (13) $$RC_{mn}=\frac{rc_{mn}+rc_{nD}}{\sum_{k=1}^{K}rc_{mk}+\sum_{k=1}^{K}rc_{nk}}$$
  where *K* is the total number of nodes encountered by a node until that instance of time, $rc_{mn}$ is the latest elapsed contact time of *m* and *n*, $rc_{nD}$ is the latest elapsed contact time of *n* and *D*, $\sum_{k=1}^{K}rc_{mk}$ is the total time elapsed since the last contact of *m* with other nodes, and $\sum_{k=1}^{K}rc_{nk}$ is the total time elapsed since the last contact of *n* with other nodes. The procedure to calculate $RC_{nD}$ is given in Algorithm 4.
- CD calculation: CD refers to the contact duration between two nodes. It is computed based on the minimum duration between a node *m* with its neighbor *n* and that between the neighbor *n* with the destination *D*. The higher the contact durability between two nodes, the higher the reliability and trustworthiness among them. It is represented by $CD_{mn}$, computed as
  (14) $$CD_{mn}=\frac{cd_{mn}+cd_{nD}}{\sum_{k=1}^{K}cd_{mk}+\sum_{k=1}^{K}cd_{nk}}$$
  where *K* is the total number of nodes encountered by a node till that instance of time, $cd_{mn}$ is the contact duration between *m* and *n*, $cd_{nD}$ is the contact duration between *n* and destination *D*, $\sum_{k=1}^{K}cd_{mk}$ represents total contact duration between *m* with all other nodes, and $\sum_{k=1}^{K}cd_{nk}$ represents total contact duration between *n* with all other nodes. The procedure to calculate $CD_{nD}$ is given in Algorithm 5.

**Algorithm 1** Beta Trust value ($BT_{mn}$).

**Input:** 

ζ,λ

**Output:** Beta Trust value ($BT_{mn}$)

1:**procedure** CALCBT(ζ, λ)2:

$\;BT_{mn}=\left(\zeta +1\right)/\left(\zeta +\lambda +2\right)$

3: return ($BT_{mn}$)4:
**end procedure**



**Algorithm 2** Encounter frequency index ($EF_{nD}$).

**Input:** n, D**Output:** Encounter frequency index ($EF_{nD}$)

1:**procedure** CALCEF(*n*,*D*)2: **for** each NId-*k* in EncounterList **do**3:    // Calculate total encounter frequency of *n* with all *k*.4:    $f_{nk}=f_{nk}+f$5:    **if** k=D **then**6:      // Calculate encounter frequency of *n* with *D*.7:      $f_{nD}=f_{nD}+f$8:    **end if**9: **end for**10:  return ($f_{nD}/f_{nk}$)11:
**end procedure**



**Algorithm 3** Packet forwarding degree ($PF_{mn}$).

**Input:** n**Output:** Packet forwarding degree ($PF_{mn}$)

1:**procedure** CALCPF(*n*)2: **for** each sid-*k* in ForwardingList **do**3:    // Calculate the total number of packets of all nodes that are forwarded by *n*.4:    $num_{nm}=num_{nm}+np$5:    **if** sid=mandrid=n **then**6:      // Calculate the total of packets of *m* that are forwarded by *n*7:      $num_{n}=num_{n}+np$8:    **end if**9: **end for**10:  return ($num_{nm}/num_{n}$)11:
**end procedure**



**Algorithm 4** Recent contact degree ($RC_{mn}$).

**Input:** m, n, D, Current time (as $t_{c}$)**Output:** Recent contact degree ($RC_{mn}$)

1:**procedure** CALCRC(*m*,*n*,*D*)2: **for** each NId-*k* in EncounterList of node *m* **do**3:    // Determine the latest elapsed contact time of *m* with *n*.4:    **if** NId=n **then**5:      $rc_{mn}=min(t_{c}-t_{le}$)6:    **end if**7:    // Determine the total time elapsed since last contact of *m* with other nodes.8:    $rc_{mk}=rc_{mk}+min(t_{c}-t_{le}$)9: **end for**10:  $\sum_{k=1}^{n}rc_{ik}=s$11:  **for** each NId-*k* in EncounterList of node *n* **do**12:     // Determine the latest elapsed contact time of *n* with destination *D*..13:     **if** k=D **then**14:       $rc_{nD}=min(t_{c}-t_{le}$)15:     **end if**16:     // Determine the total time elapsed since last contact of *n* with other nodes.17:     $rc_{nk}=rc_{nk}+min(t_{c}-t_{le}$)18:  **end for**19:  $RC_{mn}=\left\{rc_{mn}+rc_{nD}\right\}/\left\{\sum_{k=1}^{K}rc_{mk}+\sum_{k=1}^{K}rc_{nk}\right\}$20:  return $RC_{mn}$21:
**end procedure**



**Algorithm 5** Contact durability degree ($CD_{mn}$).

**Input:** m, n, D**Output:** Contact durability degree ($CD_{mn}$)

1:**procedure** CALCRC(*m*,*n*,*D*)2: **for** each NId-*k* in EncounterList of *m* **do**3:    // Determine the contact duration time of *m* with *n*.4:    **if** k=n **then**5:      $cd_{mn}=cd_{mn}+\left(t_{e}-t_{b}\right)$6:    **end if**7:    // Determine the contact duration time of *m* with all other nodes.8:    $cd_{mk}=cd_{mk}+\left(t_{e}-t_{b}\right)$9: **end for**10:  **for** each NId-*k* in EncounterList of *n* **do**11:     // Determine the contact duration time of *m* with destination *D*.12:     **if** k=D **then**13:       $cd_{nD}=cd_{nd}+\left(t_{e}-t_{b}\right)$14:     **end if**// Determine the total contact duration time of *n* with all other nodes15:     $cd_{nk}=cd_{nk}+\left(t_{e}-t_{b}\right)$16:  **end for**17:  $CD_{mn}=\left\{cd_{mn}+cd_{nD}\right\}/\left\{\sum_{k=1}^{K}cd_{mk}+\sum_{k=1}^{K}cd_{nk}\right\}$18:  return $CD_{mn}$19:
**end procedure**



By substituting the values returned by each of the above-described algorithms in Equation (8), we get the DT value $\left(DT_{mn}\right)$ of node *m* on node *n*.

### 3.3. Indirect Trust Computation

The Indirect Trust (IT) value of a node is computed by the Trustor *m* by seeking recommendations from the neighbors (referred to as Trustee nodes; an example of such node is denoted by *n*). These recommendations are obtained in the form of a Reputation Index (RI), as indicated earlier. The Indirect Trust $IT_{mn}$ is then computed by taking the average of all these reputations, i.e.,

(15) $$IT_{mn}=\frac{\sum_{k=1}^{K}RI_{mk}}{K}$$

where *K* is the total number of neighbors of *n*, and $RI_{mk}$ is the Reputation Index computed using the trust value of *m* as per the recommendation of the *k*th neighbor of *n*. The procedure to calculate $IT_{mn}$ is given in Algorithm 6.
**Algorithm 6** Calculation of Indirect Trust ($I_{mn}$).**Input:** Trustor m, Trustee n, Reputation Index (RI)**Output:** $IT_{mn}$1:**for** each neighbour, *k* of node *n* **do**2:   $RI_{nk}=\left(s_{n}^{k}+1\right)/\left(s_{n}^{k}+u_{n}^{k}+2\right)$3:**end for**4:// Compute Indirect Trust; *K* being the total neighbors5:$IT_{in}=\frac{\sum_{j=1}^{N}RI_{nk}}{K}$ =0

### 3.4. Overall Trust Computation

The overall trust of a node *n* as computed by node *m* is obtained as follows:

(16) $$OT_{mn}=\rho *DT_{mn}+\phi *IT_{mn}$$

where ρ,ϕ are constants such that ρ+ϕ=1. This value is then compared against a prescribed threshold, and nodes having a value less than that threshold are considered malicious. The procedure to calculate $OT_{mn}$ is given in Algorithm 7. The pseudo-code of our proposed CATR model is given in Algorithm 8.
**Algorithm 7** Calculation of Overall Trust Computation ($OT_{mn}$).**Input:** Direct Trust $DT_{in}$, Indirect Trust $IT_{in}$**Output:** Overall Trust Computation $OT_{in}$1:Calculate Direct Trust $DT_{in}$2:Calculate Indirect Trust $IT_{in}$3:Calculate Overall Trust using $OT_{in}=\rho *DT_{in}+\phi *IT_{in}$

**Algorithm 8** CATR routing protocol for OppIoT networks.
1:calcBT(): Method that computes and returns the Beta Trust of a node *i* on node *j* (as per Algorithm 1).2:calcEF(): Method that computes and returns the Encounter Frequency Degree between two nodes (as per Algorithm 2).3:calcPF(): Method that computes and returns the Packet Forwarding Degree of a node (as per Algorithm 3).4:calcRC(): Method that computes and returns the Recent Contact Degree between nodes (as per Algorithm 4).5:calcCD(): Method that computes and returns the Contact Durability Degree between two nodes (as per Algorithm 5).6:**for** each encountered neighbor node *n* of node *i* **do**7:   // Node *i* computes the Beta Trust of node *n*8:   $BT_{in}=calcBT\left(\right)$9:   // Node *i* computes the Encounter Frequency Degree of node *n*10:  $EF_{in}=calcEF\left(\right)$11:  // Node *i* computes the Packet Forwarding Degree of node *n*12:  $PF_{in}=calcPF\left(\right)$13:  // Node *i* computes the Recent Contact Degree of node *n*14:  $RC_{in}=calcRC\left(\right)$15:  // Node *i* computes the Contact Durability Degree of node *n*16:  $CD_{in}=calcCD\left(\right)$17:  // Computes the Direct Trust of node *i* on node *n*.18:  $DT_{in}=\omega_{1}*BT_{in}+\omega_{2}*EF_{in}+\omega_{3}*PF_{in}+\omega_{4}*RC_{in}+\omega_{5}*CD_{in}$19:  // Computes the Reputation Index for node *n* based on reputations from neighbors of node *n*.20:  **for** each neighbour *k* of node *n* **do**21:    $RI_{nk}=\left(s_{n}^{k}+1\right)/\left(s_{n}^{k}+u_{n}^{k}+2\right)$22:  **end for**23:  // Compute the Indirect Trust x, *K* being the total neighbors (as per Algorithm 6)24:  $IT_{in}=\frac{\sum_{j=1}^{N}RI_{nk}}{K}$25:  // Compute the Overall Trust (OT) (as per Algorithm 7)26:  $OT_{in}=\rho *DT_{in}+\phi *IT_{in}$27:  // Compare OT with a threshold trust value τ28:  **if** $OT_{in}\geq \tau$ **then**29:    Forward the message from node *i* to node *n*30:  **end if**31:
**end for**



### 3.5. Complexity Analysis

In the outer loop, the algorithm iterates over all encountered neighbor nodes of node *i*. Let *N* represent the number of neighbors of node *i*. The time complexity of the outer loop is O(*N*). Each method (calcBT(), calcEF(), calcPF(), calcRC(), calcCD()) is invoked once per neighbor. So, the time complexity for all trust metric calculations per neighbor is O(1).

In the inner loop, for each neighbor *n*, the algorithm iterates over all neighbors of $K_{n}$, which represents the number of neighbors of *n*. The combined time complexity for all neighbors is $\sum_{n=1}^{N}K_{n}$.

The threshold comparison and potential forwarding are O(1) per neighbor. The total time complexity is the sum of the complexities of all operations is O(*N*) + O(1) + O($\sum_{n=1}^{N}K_{n}$).

In the worst case, assuming each node *i* has *N* neighbors and each of those neighbors has an average of *K* neighbors, then the total time complexity consumed is O(*N* * K). If *K* scales linearly with *N*, the complexity becomes O($N^{2}$).

## 4. Performance Evaluation

We have evaluated our proposed CATR routing protocol for OppIoT using an Opportunistic Network Environment (ONE) simulator [5] and compared its performance against that of the Epidemic [6], BTRES [7], and PPHB+ [8] protocols, chosen as the benchmark, in terms of the following performance metrics:

- Average latency: This is the amount of time a message needs to travel across the network to reach its intended destination. It is computed as the time difference between creating and delivering messages.
- Message drop rate: This is the rate at which messages are lost during data transmission. The smaller the number of dropped packets, the higher the efficiency of the model.
- Overhead ratio: This is the proportion of additional communication or computational resources consumed apart from the essential data transmission requirements. A routing model having less amount of overhead is considered as efficient.
- Hop count average: This is the mean number of nodes the message passes through between the sender and destination nodes. A decreased hop count corresponds to an increased efficiency.
- Message delivery probability: This is the probability of receiving the message accurately at the destination node. A routing model with a higher message delivery probability is considered efficient.

The weights for evaluating the metrics Beta Trust Degree, Encounter Frequency, Packet Forwarding Degree, Recent Contact Degree, and Contact Durability Degree were determined through extensive simulations conducted using the ONE simulator. By systematically varying the weight values of these parameters, we optimized the configuration to achieve the best delivery probability.

Four parameters are modified during our evaluations: number of nodes: {66, 96, 126, 156, 186}); time to live (TTL): {300, 350, 400, 450, 500} min; message generation interval: {5–15, 15–25, 25–35, 35–45, 45–55} s; and buffer space: {5, 10, 15, 20, 25} MB. Also, the general parameters used in our simulations comprise 96 mobile nodes split into 6 groups: 1 group of vehicles with 30 nodes, 3 groups of trams with 2 nodes each, and 2 groups of pedestrians with 30 nodes each. With a buffer size of 10 MB, each pedestrian node moves between 0.5 and 1.5 m per second. With a buffer capacity of 50 MB, the tram group’s nodes move at a pace of 7–10 m/s. The speed of car group nodes is 2.7–13.9 m/s, each having a buffer size of 10 MB. Each node generates a message within an interval of 25 to 35 s of size 500 KB to 1 MB and 300 min TTL (time to live) for the message. The other simulation parameters are represented in Table 3.

Based on the aforementioned performance metrics, our model’s effectiveness compared to that of the chosen benchmark models, is assessed. We have tested the proposed model using the real CRAWDAD dataset Cambridge/haggle (29 May 2009) [20]. The simulation results are captured in Figure 2, Figure 3, Figure 4, Figure 5, Figure 6, Figure 7, Figure 8, Figure 9, Figure 10 and Figure 11.

**Figure 2:**
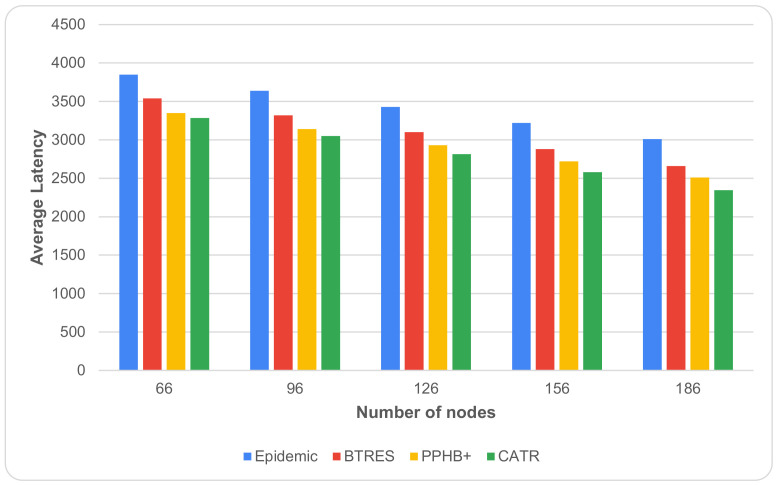
Average latency vs. number of nodes.

Figure 2 shows the effects of the variations in the average latency as the number of nodes increases from 66 to 186. There is a decrease in average latency as the number of nodes rises because more and more nodes become available in the network. With an increase in the number of nodes, it is observed that CATR outperforms the Epidemic, BTRES, and PPHB+ protocols by 24%, 25%, and 13%, respectively, in terms of average latency.

**Figure 3:**
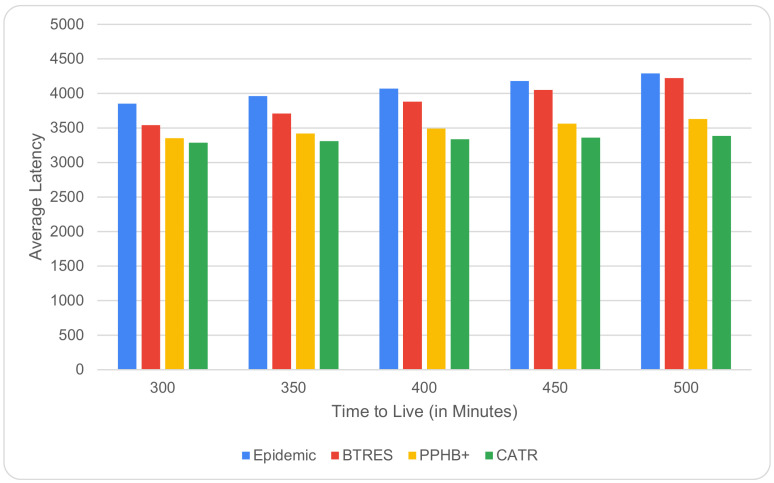
Average latency vs. TTL.

Figure 3 shows how an increase in TTL from 300 to 500 min affects the average latency. When a message’s TTL value rises, that message can remain in the nodes’ buffer for an extended period of time, lowering the message’s average latency. As can be observed, in terms of the average latency on increasing the TTL, our proposed CATR protocol performs better than the Epidemic, BTRES, and PPHB+ protocols by 8%, 18%, and 6%, respectively.

**Figure 4:**
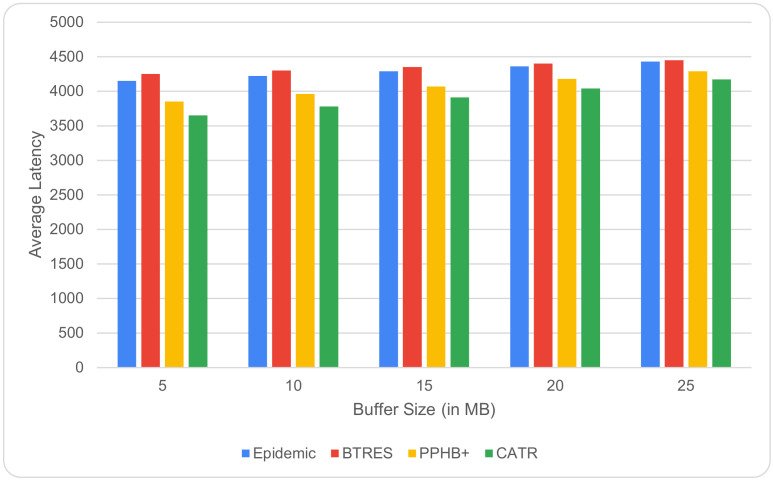
Average latency vs. buffer size.

A node can keep the data packets for a longer period of time when the buffer size is increased from 5 MB to 25 MB, which ultimately results in an increase in latency. This relation is shown in Figure 4, where it is evident that, with an increase in the buffer size, our proposed CATR protocol outperforms the Epidemic, BTRES, and PPHB+ protocols in terms of average latency by 17%, 25%, and 8%, respectively.

**Figure 5:**
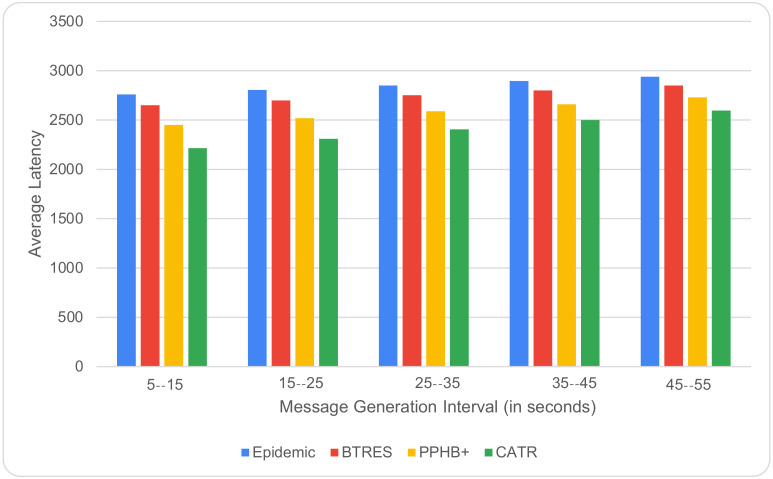
Average latency vs. message generation interval.

Figure 5 shows the relation between the message generation interval and average latency, where it is found that with an increase in the interval from 5 to 15 to 45–55 s, the average latency also gradually rises for the studied protocols. However, this value is much lower for our proposed CATR model compared to what is obtained for the other studied models, with CATR performing 17%, 23%, and 9% better than the Epidemic, BTRES, and PPHB+ protocols, respectively.

**Figure 6:**
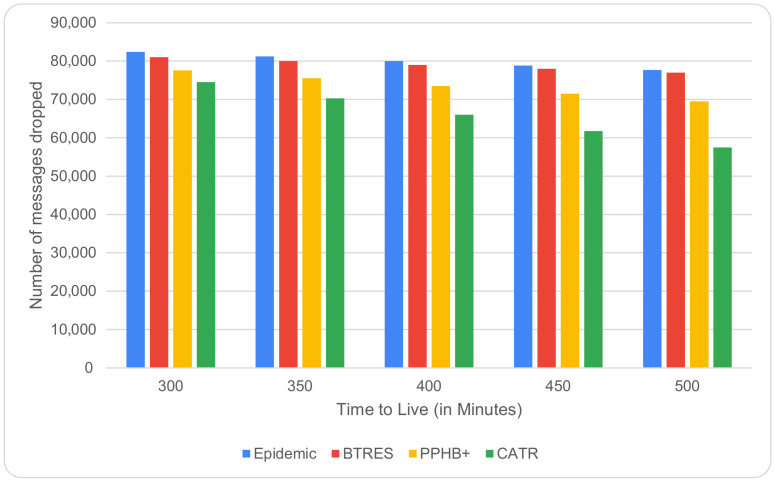
Messages dropped vs. TTL.

In Figure 6, it is found that as the TTL value increases from 300 to 500, there is a significant improvement in our CATR model in terms of messages dropped. This is due to the fact that a higher value of TTL implies that the message can remain in the network for a longer period of time. In terms of the average number of dropped messages, our CATR scheme is 24%, 12%, and 14% better than the Epidemic, BTRES, and PPHB+ protocols, respectively.

**Figure 7:**
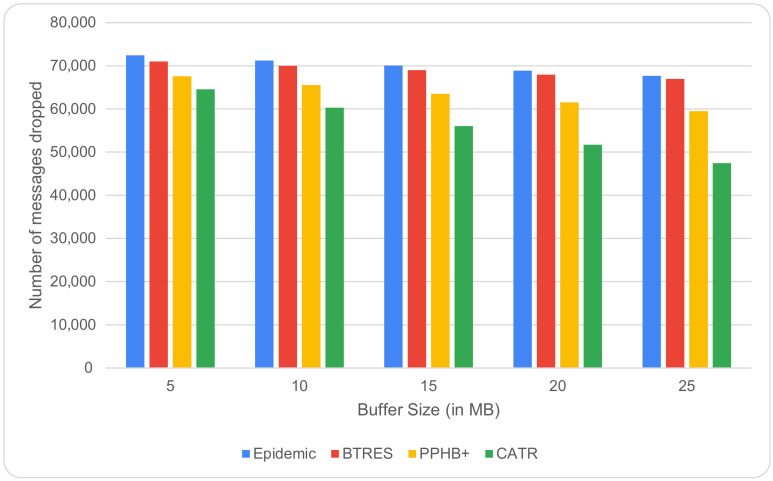
Messages dropped vs. buffer size.

The relationship between the number of dropped messages and buffer size is shown in Figure 7. It can be observed that when the buffer size is increased from 5 MB to 25 MB, the average number of dropped messages is significantly lower than for Epidemic, BTRES, and PPHB+, improving the performance by 23%, 18%, and 16%, respectively. This is because a node’s larger buffer allows it to store a message for longer periods of time, which lowers the amount of messages dropped.

**Figure 8:**
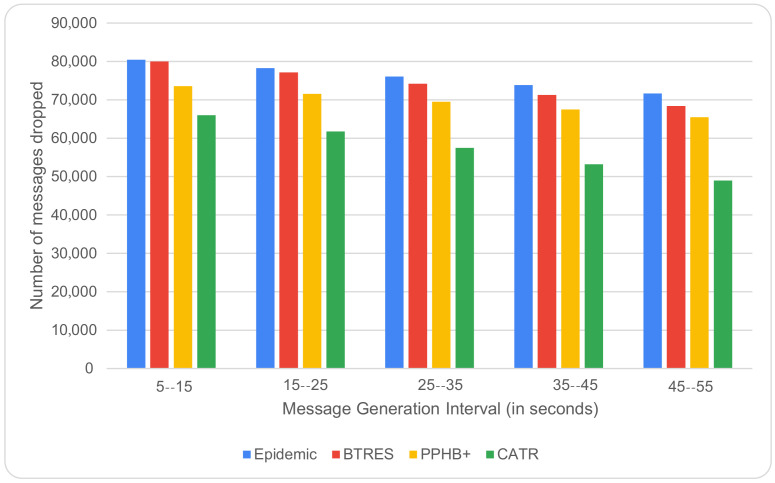
Messages dropped vs. message generation interval.

The impact of varying the message production interval on the message dropping rate is shown in Figure 8. This rate eventually decreases by 23%, 16%, and 17% compared to Epidemic, BTRES, and PPHB+, respectively, as the message generation interval is increased from 5 to 15 to 45–55. This is because an increase in the message generation interval allows a node to store data packets in its buffer for a longer period of time before any new packet is generated.

**Figure 9:**
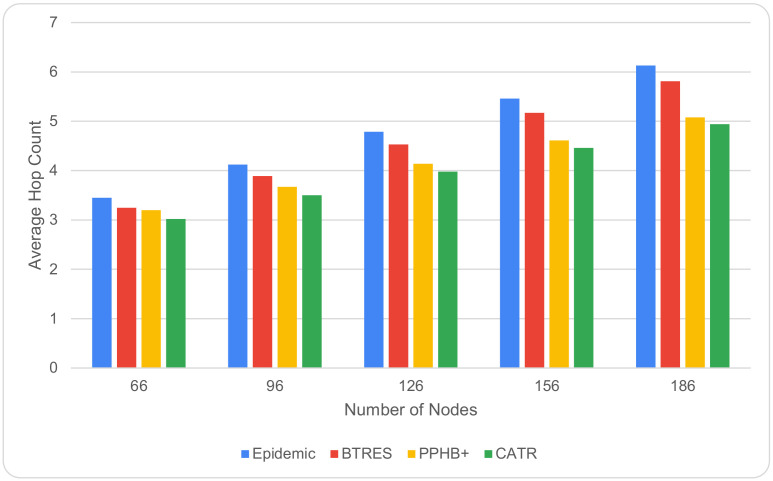
Hop count Vs. number of nodes.

Figure 9 shows how the number of nodes and hop count are related. It can be shown that for all studied models, the number of hop counts increases as the node count rises from 66 to 186. This is due to the fact that as there are more nodes in the network, there is an increasing number of nodes that are available. However, compared to Epidemic, BTRES, and PPHB+, our proposed CATR model requires a significantly smaller number of hops, improving the performance by 34%, 14%, and 10%, respectively.

**Figure 10:**
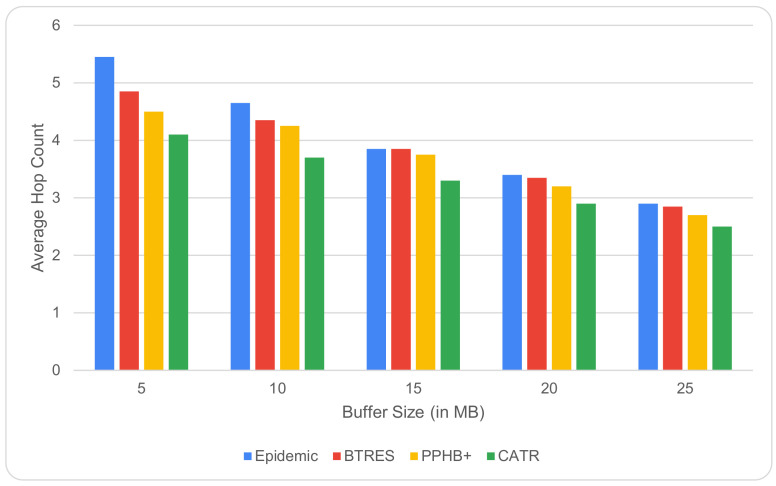
Hop count vs. buffer size.

The impact of changing the hop count as the buffer size increases from 5 to 25 MB is depicted in Figure 10. For every model, the hop count progressively drops as a node’s buffer size grows. It is explained by the fact that the node can store more and more messages as the buffer size increases, which eventually results in fewer hops. Compared to Epidemic, BTRES, and PPHB+, the proposed CATR’s value is significantly lower, which improves its performance by 29%, 10%, and 19%, respectively.

**Figure 11:**
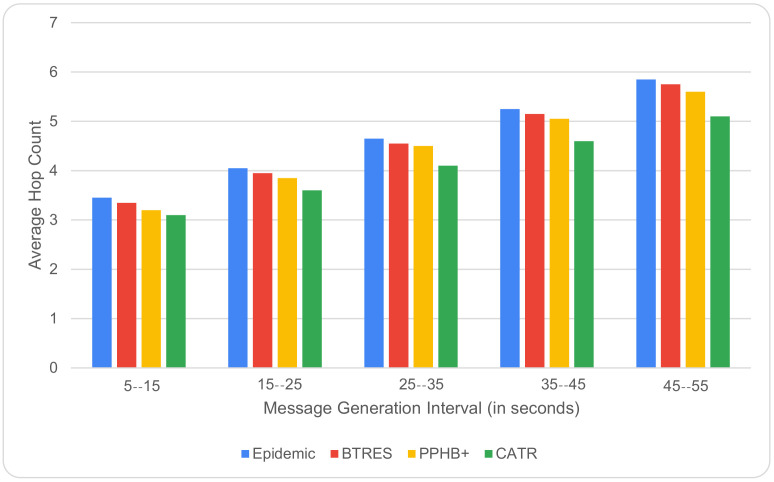
Average hop count vs. message generation interval.

Figure 11 depicts the relation between the number of hops and the message generation interval, where it is clear that with an increase in the message generation interval from 5 to 15 to 45–55, the number of hops required to propagate a message gradually increases. However, this increase is much lower in the case of our proposed CATR, hence performing better than Epidemic, BTRES, and PPHB+ by 30%, 14%, and 9%, respectively. The fact is that when there is an increase in the message generation interval, a node can store data packets in its buffer for a longer duration of time before generating any new packets, and this eventually leads to a decrease in the average hop count for that message.

## 5. Conclusions

In this paper, a Context-Aware Trust and Reputation Routing protocol for OppIoT networks (called CATR) is proposed, in which the trust values of nodes are computed dynamically. In such a scheme, before forwarding any data packet to its neighbor node, a source node computes the trust of that node in two phases—Direct Trust and Indirect Trust. These trust values serve as key parameters in deciding on suitable relay nodes to carry the message from its source to its eventual destination. Simulation experiments conducted using the ONE simulator demonstrate that CATR outperforms the Epidemic protocol, the beta-based Trust and Reputation Evaluation System (BTRES), and the secure and privacy-preserving scheme structure in opportunistic networks (PPHB+). Specifically, CATR achieves improvements of 22%, 15%, and 9% in terms of average latency, number of messages dropped, and average hop count, respectively. These results hold under varying conditions, including the number of nodes, buffer size, time to live (TTL), and message generation intervals. In a future study, this model can be extended by incorporating a mechanism to enhance the performance metrics.

## Figures and Tables

**Table 1:** Notations.

Notations	Meaning
*N*	Number of nodes in the network
*n*	Neighbor node
*S*	A message’s source node
*D*	Node to which a message is destined
*s*	Sender node
*r*	Receiver node
*m*	Node that computes the trust value of a node (Trustor node)
*M*	Messages generated by a node
ζ	Number of successful encounters between two nodes
$\omega_{k}$	Weights for different constants, used in Equation (8), where k = 1, 2, 3..
λ	Number of unsuccessful encounters between two nodes
$BT_{mn}$	Beta Trust of node *n* as computed as node *m*
$EF_{nD}$	Encounter Frequency Degree of node *n* with *D*
$PF_{mn}$	Packet Forwarding Degree of *n* as computed by *m*
$RC_{mn}$	Recent Contact Degree of *n* as computed by *m*
$CD_{mn}$	Contact Durability Degree of *n* as computed by *m*
$DT_{mn}$	Direct Trust of *n* as computed by *m*
$IT_{mn}$	Indirect Trust of *n* as computed by *m*
$OT_{mn}$	Overall Trust of *n* as computed by *m*
*K*	Total number of nodes encountered by node *n* till that instance of time.
*p*	Probability of receiving an untrusted signal from an untrusted node
*q*	Probability of receiving a trusted signal from a trusted node are considered

**Table 2:** Hashmaps.

Hashmaps	Significance
EncounterList	Hashmap consisting of fields $<NId,f,\zeta ,\lambda ,t_{be},t_{ee},t_{e}>$ that store records of encounter details of a node with other nodes in the network. These fields correspond to node identifier, encounter frequency, successful encounter, unsuccessful encounter, encounter begin time, encounter end time, and total encounter duration, respectively.
ForwardingList	This hashmap stores records of packet forwarding using 3 fields <sid,rid,np> that correspond, respectively, to sender ID, receiver ID, and number of packets forwarded from sid to rid.
TrustDegree	Stores the trust value of nodes in two fields $<Node,T_{Node}>$, where Node represents a node for which its trust value is computed and $T_{Node}$ is the trust value of that node.

**Table 3:** Simulating parameters.

Parameters	Values
Simulation area	4500 × 3400 m
Simulation time	100,000 s
Movement model	Shortest-path-map-based movement model
Interface for communication	Bluetooth
Transmission diameter	20 m
Transmission speed	250 Kbps
High-speed interface transmission range	1500 m
High-speed interface transmission speed	10 Mbps
TTL (time to live)	300 min
Message size	500 KB to 1 MB
Message creation interval	25 to 35 s

## Data Availability

Data will be made available on request.
